# Evolutionary and Transcriptomic Analyses of the Plant TPST-Sulfated Peptides System, with Insights from Woody *Liriodendron chinense*

**DOI:** 10.3390/plants15071115

**Published:** 2026-04-04

**Authors:** Yu Liu, Kaiyue Hong, Teja Manda, Xiangyang Hu, Liming Yang

**Affiliations:** 1 State Key Laboratory of Tree Genetics and Breeding, College of Life Sciences, Nanjing Forestry University, Nanjing 210037, China; ly1833@njfu.edu.cn (Y.L.); awzh18252056628@163.com (K.H.); teja.manda@njfu.edu.cn (T.M.); 2 Shanghai Key Laboratory of Bio-Energy Crops, School of Life Sciences, Shanghai University, Shanghai 200444, China

**Keywords:** sulfated peptides, tyrosylprotein sulfotransferase (TPST), phylogenetic analysis, molecular docking, transcriptional regulation

## Abstract

Sulfated peptides, such as PSK, PSY, CIF, and RGF, are crucial regulators of plant growth, development, and stress responses, with their activity dependent on post-translational tyrosine sulfation by tyrosylprotein sulfotransferase (TPST). This study explores the evolutionary history and the interaction mechanisms between TPST and sulfated peptides in plants. Systematic analyses of multi-species genomes show that TPST can be traced back to the chlorophyte lineage, whereas PSK, a sulfated peptide, appears to have emerged in gymnosperms. TPST is evolutionarily conserved, typically present in low copy numbers across plant lineages, while its peptide substrates have expanded in angiosperms. In *Liriodendron chinense*, TPST-sulfated peptide gene promoters are enriched with *cis*-regulatory elements linked to abscisic acid, gibberellin responsiveness, and anaerobic induction. Synteny analyses revealed collinearity between sulfated peptide genes in *L. chinense*, *Magnolia biondii*, *Arabidopsis thaliana*, and *Populus trichocarpa*, but not with *Oryza sativa*. Molecular docking identified key TPST-PSK interaction sites in the sulfotransferase domain, with several critical residues facilitating binding. Transcriptomic and co-expression network analyses revealed that *LcTPST* was expressed at lower levels than its peptide precursor genes, while *LcPSK2* remained highly expressed after the torpedo stage of somatic embryogenesis. Stress conditions significantly increased PSK-associated module connectivity, enriched in transcription factors such as WRKY, bHLH, bZIP, and MADS. This study provides insights into the evolutionary, structural, and regulatory aspects of the TPST-sulfated peptide system in plants.

## 1. Introduction

Plants produce a diverse repertoire of peptide hormones, including canonical secreted peptides as well as non-canonical peptides (or those lacking a classical signal peptide) [1]. These nuclear genome–encoded peptide hormones typically undergo proteolytic processing of their precursor proteins and post-translational modifications to generate biologically active mature peptides [1,2]. Recent studies have shown that small signaling peptides not only participate in cell fate reprogramming during plant regeneration but also play important roles in stress adaptation. Existing reviews have indicated that distinct peptide–receptor pathways are involved in key morphogenetic processes such as shoot regeneration, root regeneration, and somatic embryogenesis. In *Brassica napus*, genome-wide analysis of the *PSK* gene family together with treatment experiments further showed that some *BnPSK* members are induced by drought and ABA, accompanied by increased antioxidant enzyme activity and changes in ROS signaling, suggesting that *PSK* may participate in drought responses through ABA-related redox regulation. Sulfated peptides often function in a tissue- or cell type-specific manner [3,4]. In *A. thaliana*, RGF peptides are required for the maintenance of the root apical stem cell niche; CIF peptides function in stele-to-endodermis signaling for Casparian strip formation; TWS1 functions at the embryo-endosperm interface during seed development. PSK signaling is involved in pollen tube growth and funicular pollen tube guidance. These examples support the view that sulfated peptides act mainly through spatially restricted cell-to-cell communication [1,5,6,7].

Sulfated peptides constitute a class of secreted peptides, including PSKs (PHYTOSULFOKINEs), PSYs (PLANT PEPTIDE CONTAINING SULFATED TYROSINE), CIFs (CASPARIAN STRIP INTEGRITY FACTORs), and RGFs (ROOT MERISTEM GROWTH FATORs) [8]. These peptides play crucial roles in multiple aspects of plant growth and development, as well as in immune responses [9]. For example, PSK promotes cell division and tissue regeneration, stimulates primary root elongation, and facilitates the formation of lateral and adventitious roots [10,11]. In *A. thaliana*, loss-of-function mutants lacking *psk* or *tpst* exhibit reduced root meristem activity and a dwarf phenotype [11,12]. During somatic embryogenesis, exogenous PSK application increases the likelihood that non-embryogenic callus acquires embryogenic competence, enhances somatic embryo induction, and supports continued embryo development [13,14]. The PSK–PSKR pathway has been implicated in species-specific regulation of growth, reproduction, and defense. In *A. thaliana*, PSK signaling is required for pollen tube growth and funicular pollen tube guidance, whereas in *Solanum lycopersicum* L., PSK perception by PSKR1 elevates cytosolic Ca^2+^ and activates auxin-dependent immunity against *Botrytis cinerea*. In addition, studies in *A. thaliana* indicate that PSKR1 contributes to growth defense trade-offs by modulating salicylic acid-associated immune responses. [11,15,16,17,18]. Studies have shown that, in the primary root of *A. thaliana*, PSY1 promotes root growth mainly by regulating cell elongation and mature cell size in the elongation and differentiation zones [19]. The PSY/PSYR signaling module functions at multiple stages of growth and development, with certain roles being particularly associated with early developmental phases [9]. In addition, PSY peptides are involved in plant responses to salt stress and drought stress [9]. CIF peptides were first identified in *A. thaliana* and are closely associated with the integrity of the endodermal Casparian strip [6]. In *A. thaliana*, *CIFs* are produced in the root stele and perceived by SGN3/GSO1 in the endodermis, where they promote proper formation and integrity of the endodermal Casparian strip, a major inner apoplastic diffusion barrier [6,20,21]. RGFs (also known as GLV/RGF/CLEL peptides) are key factors required for the maintenance of the root apical stem cell niche; in *A. thaliana*, rgf multi-gene mutants display a reduced root meristem and impaired root growth [22,23].

Tyrosylprotein sulfotransferase (TPST) catalyzes the transfer of a sulfate group from 3′-phosphate-5′-phosphosulfate (PAPS) to tyrosine residues in diverse proteins or peptide substrates, thereby generating tyrosine-sulfated products [24]. This post-translational modification plays an important role in plants [12]. In *A. thaliana*, TPST is responsible for converting peptides such as PSK, PSY, RGF, and CIF into their bioactive forms through tyrosine sulfation [12,24]. These bioactive peptides are broadly conserved across plant species and play essential roles by regulating growth, development, and stress responses [8,9]. In *A. thaliana*, *tpst* mutants exhibit multiple developmental defects. In roots, defective activation of sulfated peptides, particularly CIFs, compromises Casparian strip integrity, thereby impairing the selective barrier required to maintain a signal-rich root meristematic environment and consequently reducing meristem activity and primary root growth. In addition, failure to properly activate other sulfated peptides, including PSK, PSY, and RGF, contributes to defects in reproductive development such as abnormal pollen and ovule formation [2,9].

*Liriodendron* belongs to Magnoliaceae and the magnoliids [25]. As magnoliids are regarded as one of the earliest-diverging lineages within the Mesangiospermae [25], *Liriodendron* occupies a pivotal position for elucidating early angiosperm evolution [25]. The key phylogenetic position of *L. chinense* in angiosperm evolution makes it an ideal model for investigating the interaction mechanisms of the plant TPST-sulfated peptide system, and its genomic features provide a unique perspective for exploring the roles of sulfated peptides in plant growth, development, and stress adaptation [25].

In this study, we detected TPST and its catalyzed sulfated peptides, including PSK, PSY, CIF, and RGF gene families, from a broad range of green plant genomes to infer the origin of the TPST-sulfated peptide system. Against the diverse genetic backgrounds of land plants, we further characterized the evolutionary model of TPST and its sulfated peptide substrates. The physicochemical properties of each family member were analyzed to explore structural-functional associations and divergences. Based on *cis*-regulatory element analyses of promoter regions from TPST–sulfated peptide system genes in *L. chinense*, we assessed their potential regulation by environmental stresses and phytohormone signaling. Molecular docking was employed to investigate the binding conformations between TPST and three peptide substrates (PSK/PSY/CIF), to identify key interacting residues and infer determinants of substrate specificity. In addition, transcriptome datasets were used to profile the expression patterns of *TPST*, *PSK*, *PSY*, and *CIF* genes in *L. chinense* across somatic embryogenesis, multiple organs, and diverse stress treatments; these results, integrated with co-expression network analyses and transcription factor screening, provide candidate genes for future functional validation. Through multi-layer integrative analyses, this study aims to elucidate the evolutionary history and structural recognition mechanisms of the TPST-peptide system in plants. Using the early-diverging mesangiosperm *L. chinense* as a focal species, we systematically investigated the structure and evolution of TPST and the PSK, PSY, and CIF gene families, providing a foundation for understanding their interaction mechanisms and functional importance in angiosperms.

## 2. Results

### 2.1. Lineage-Specific Distribution and Evolutionary Patterns of the TPST Gene Family and Sulfated-Peptide Genes

**Figure 1:**
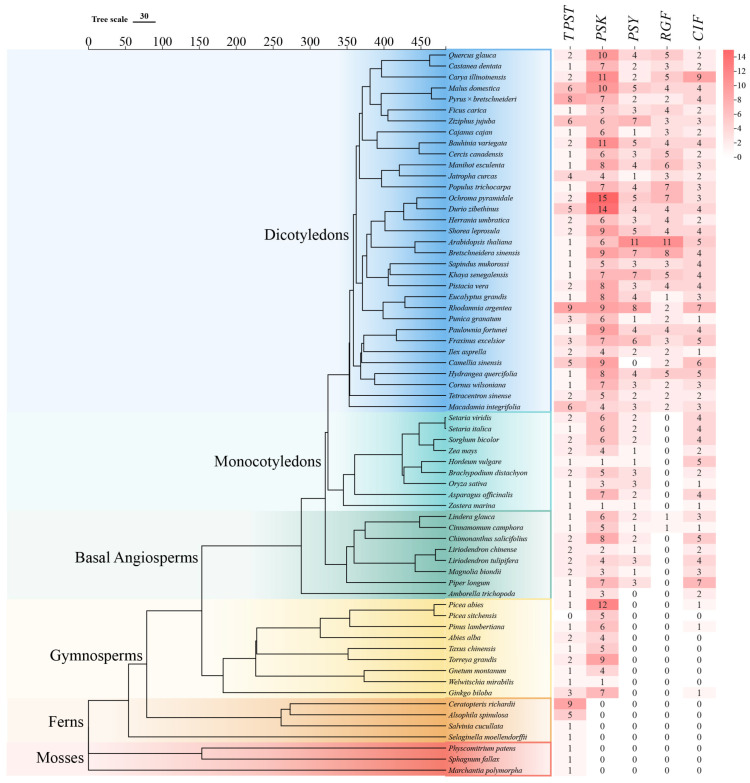
Genome-wide identifications of tyrosylprotein sulfotransferase (TPST) and its sulfated peptide substrates (PSK, PSY, RGF, and CIF) across 66 plant species. (**Left**): species phylogenetic tree of the 66 plants. (**Right**): gene family sizes (copy numbers) of TPST, PSK, PSY, RGF, and CIF.

Broad phylogenomic screening revealed the presence of TPST family members in 60 chlorophyte species (Supplementary Table S1), consistent with previous reports that TPST can be traced back to the chlorophyte stage (e.g., *Ostreococcus lucimarinus*) [26]. In gymnosperms, PSK and CIF family members were identified, representing the earliest occurrences among the four sulfated peptide families. PSKs showed a broader distribution and higher copy numbers across the nine gymnosperm species examined, whereas CIFs were detected only in *Picea abies*, *Pinus lambertiana*, and *Ginkgo biloba*, with a single copy in each species. PSYs first appeared in basal angiosperms and subsequently underwent moderate copy-number expansion during plant evolution. Although RGFs were initially detected in gymnosperms, they were found only in two Lauraceae species (*Lindera glauca* and *Cinnamomum camphora*) and were not detected in the nine monocot species analyzed. Overall, TPST is maintained as a low-copy gene (typically <4 copies) in most species, with copy numbers exceeding five observed only in a few taxa (e.g., *Ceratopteris richardii*, *Rhodamnia argentea*, and *Pyrus* × *bretschneideri*) (Figure 1).

### 2.2. Phylogenetic Analyses of TPST, PSK, PSY, RGF, and CIF

**Figure 2:**
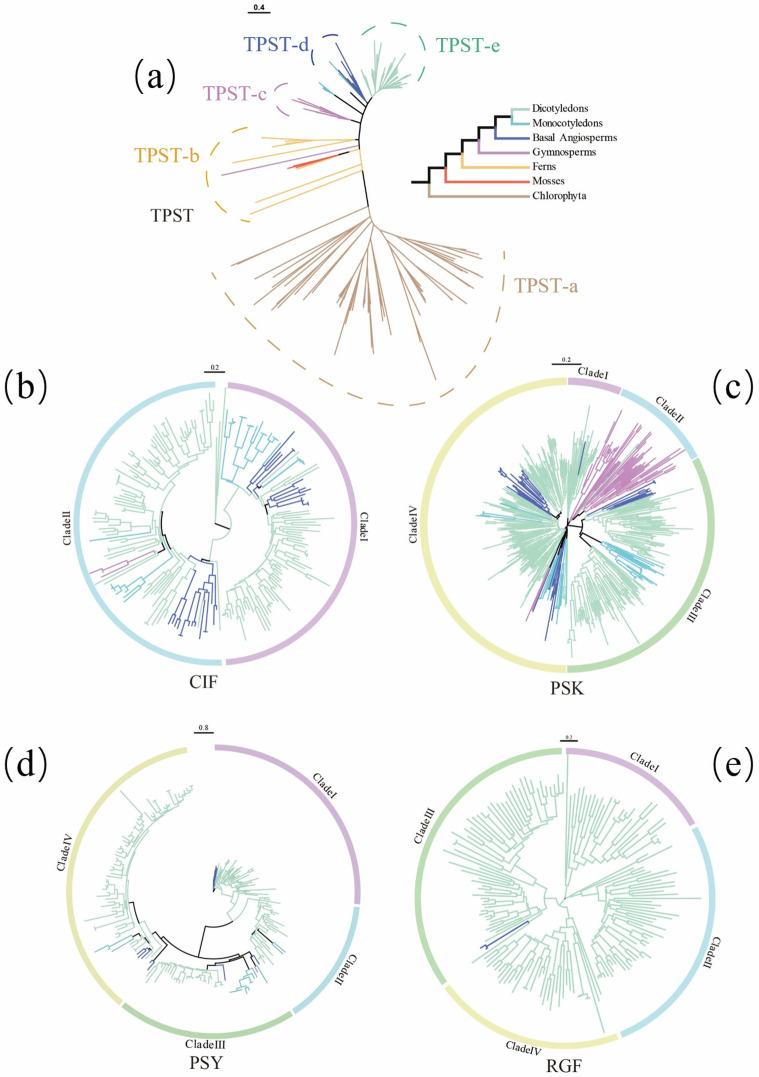
Phylogenetic analysis of plant tyrosylprotein sulfotransferase (TPST) and its sulfated peptide substrates (PSK, PSY, RGF, and CIF). (**a**) Neighbor-joining (NJ) tree topology of the TPST gene family. Genes are color-coded by lineage to distinguish eudicots, monocots, basal angiosperms, gymnosperms, ferns, bryophytes, and chlorophytes. (**b**–**e**) NJ tree topologies of the CIF, PSK, PSY, and RGF gene families. Branch colors follow the same scheme as in panel (**a**).

Phylogenetic analyses showed that the *TPST* gene family can be classified into five clades, designated TPST-a, TPST-b, TPST-c, TPST-d, and TPST-e. Chlorophyte TPST members were predominantly clustered within the TPST-a clade, whereas eudicot TPST members were largely enriched in the TPST-e clade. Overall, TPST sequences from each major plant lineage tended to form lineage-specific clusters in the tree topology (Figure 2a). In contrast, *CIF*, *PSK*, *PSY*, and *RGF* comprised two, four, four, and four clades, respectively. In the CIF phylogeny, the three gymnosperm members formed a distinct sub-branch (Figure 2b). Most gymnosperm PSKs were concentrated in Clade II (Figure 2c). As the most recently emerged sulfated peptide families, PSY and RGF showed low copy numbers and tight topological clustering in basal angiosperms (Figure 2d,e). Notably, RGF represents the latest-appearing family among the four sulfated peptide groups. The two RGF members identified from the two Lauraceae species (*Lindera glauca* and *Cinnamomum camphora*) clustered into a single sub-branch, indicating a pronounced lineage-restricted pattern.

### 2.3. Physicochemical Characterization of TPST, PSK, PSY, RGF, and CIF

**Figure 3:**
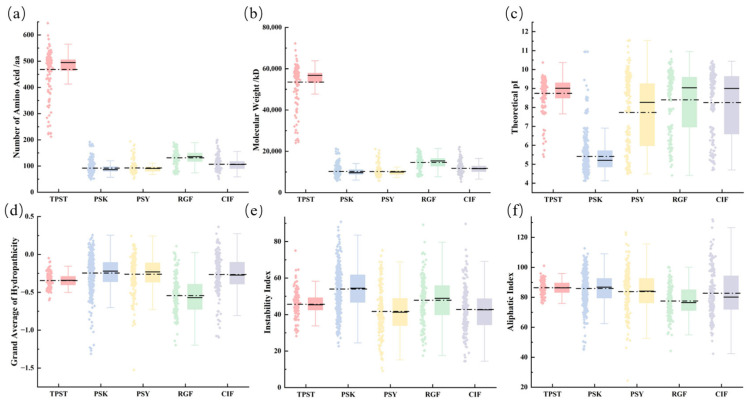
Physicochemical properties of proteins in the TPST, PSK, PSY, RGF, and CIF families. (**a**) Amino acid length. (**b**) Molecular weight. (**c**) Theoretical pI. (**d**) Grand average of hydropathicity (GRAVY). (**e**) Instability index. (**f**) Aliphatic index. The solid line indicates the median, and the dotted line indicates the mean.

As the key enzyme catalyzing tyrosine sulfation, TPST exhibits a relatively large molecular weight (~45–70 kDa) and a long amino acid sequence (~400–600 aa). It is overall basic (pI ≈ 8–9) and strongly hydrophilic, consistent with its catalytic role in the Golgi apparatus. In contrast, its peptide substrates-PSK, PSY, RGF, and CIF-are typically small precursor peptides (<150 aa; <20 kDa), with theoretical pI values spanning ~5–11. PSKs are markedly acidic, whereas PSY/RGF/CIF precursors are predominantly basic and show family-specific differences in stability and aliphatic index (Figure 3).

### 2.4. Cis-Regulatory Element Analysis of Gene Family Members in L. chinense

**Figure 4:**
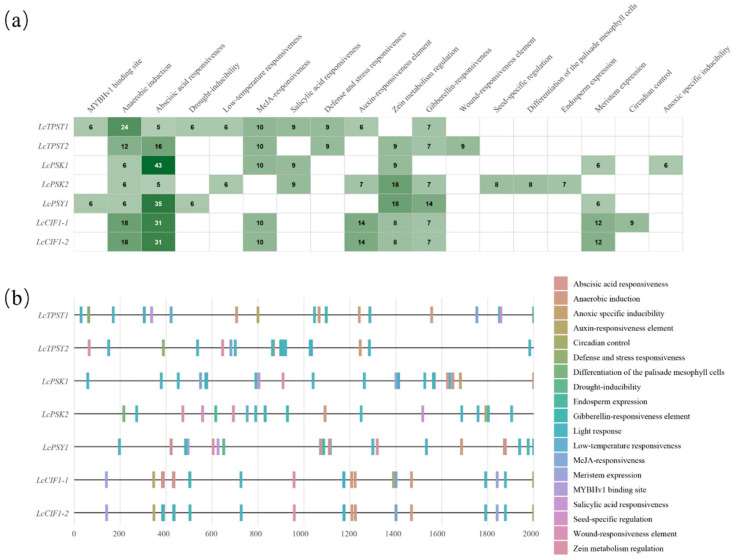
*Cis*-regulatory elements in the promoters of *L. chinense TPST*, *PSK*, *PSY*, and *CIF* genes. (**a**) Summary of the types and numbers of *cis*-regulatory elements identified in the promoter regions of *TPST*, *PSK*, *PSY*, and *CIF* in *L. chinense*. (**b**) Distribution of *cis*-regulatory elements within the 2 kb promoter sequences of *L. chinense TPST*, *PSK*, *PSY*, and *CIF* genes. Different *cis*-elements are represented by colored rectangles positioned at their corresponding sites along the promoters.

*Cis*-element profiling was performed using a 2 kb promoter region of seven *L*. *chinense* genes (*LcTPST1*, *LcTPST2*, *LcPSK1*, *LcPSK2*, *LcPSY*, *LcCIF1-1*, and *LcCIF1-2*) (Figure 4a,b). At the quantitative level (Figure 4a), these promoters were generally enriched for *cis*-regulatory elements associated with hormone responsiveness (ABA, IAA, GA, SA, and JA) and stress responses (drought, low temperature, anaerobic induction, wounding, and defense). The promoters of *LcTPST1*/*LcTPST2* were characterized mainly by anaerobic induction- and ABA-responsive elements, whereas *LcPSK1* showed the strongest enrichment for ABA-responsive elements. Notably, the *LcPSY* promoter contained particularly high counts of auxin- and gibberellin-responsive elements (“Auxin-responsiveness element” and “Gibberellin-responsiveness element”, 18 and 14, respectively). In addition to stress- and hormone-related motifs, *LcPSK2* also harbored several development-associated signals, including “Seed-specific regulation”, “Differentiation of the palisade mesophyll cells”, and “Endosperm expression” (8, 8, and 7, respectively) (Figure 4a,b).

The two tandemly duplicated *CIF* genes showed broadly similar *cis*-element compositions (both containing ABA-, IAA-, GA-, SA/JA-responsive elements as well as low-temperature- and defense-related motifs), yet differences were also evident: a circadian control element was uniquely detected in *LcCIF1-2*. This divergence may reflect de novo insertions and other promoter mutations following duplication, together with local chromatin-context differences, which could confer circadian-dependent regulation of gene expression (Figure 4a).

Overall, the 2 kb promoter regions of *L. chinense TPST*, *PSK*, *PSY*, and *CIF* genes broadly integrate *cis*-regulatory elements related to phytohormone signaling (ABA/IAA/GA/SA/JA) and stress responses (drought, low temperature, anaerobic induction, and wounding/defense). The *LcTPST* promoters were biased toward anaerobic- and ABA-responsive motifs, the *LcPSK1* promoter showed pronounced enrichment of ABA-responsive elements, and the *LcPSY* promoter was enriched for auxin- and gibberellin-related elements. The two *LcCIF* copies exhibited an overall conserved *cis*-element framework, with only minor differences in specific motifs (Figure 4a).

### 2.5. Chromosomal Localization and Synteny Analysis

**Figure 5:**
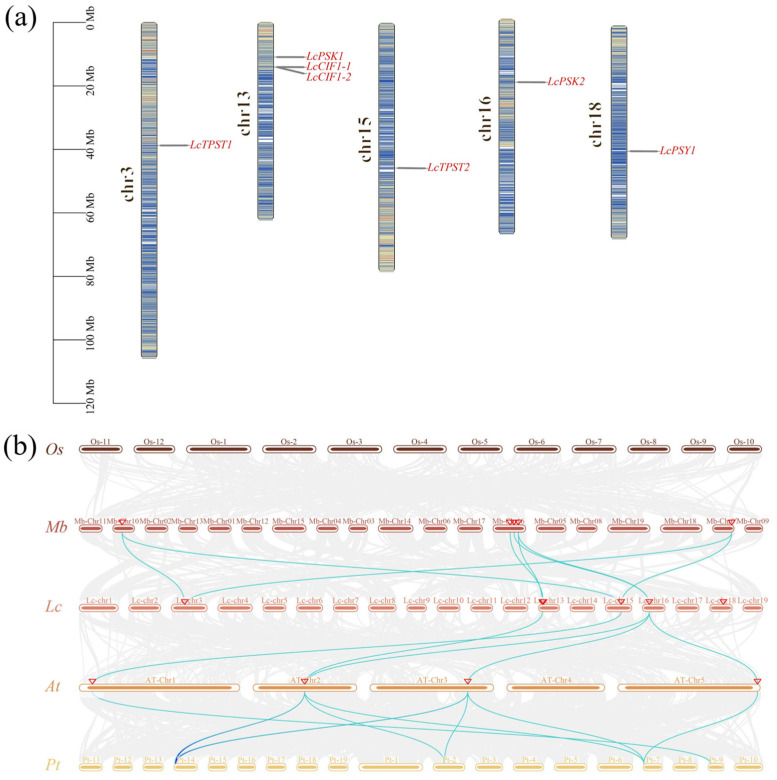
Chromosomal distribution and cross-species synteny analysis of *TPST*, *PSK*, *PSY*, and *CIF*. (**a**) Physical locations of *TPST*, *PSK*, *PSY*, and *CIF* genes on the chromosomes of *L. chinense;* gene names are annotated to the right of each chromosome. (**b**) Syntenic comparison of *TPST*, *PSK*, *PSY*, and *CIF* loci among *O. sativa*, *M. biondii*, *L. chinense*, *A. thaliana*, and *P. trichocarpa*. Gray lines indicate collinear blocks of annotated genes across the genomes, whereas highlighted green links specifically trace conserved orthologous pairs of *TPST*, *PSK*, *PSY*, and *CIF*. The red inverted triangles in *L. chinense* mark, from left to right, *LcTPST1* (Chr3), *LcPSK1* (Chr13), *LcCIF1-1* (Chr13), *LcTPST2* (Chr15), *LcPSK2* (Chr16), and *LcPSY1* (Chr18). Chr, chromosome.

These seven *TPST*, *PSK*, *PSY*, and *CIF* genes in the *L. chinense* genome are distributed across five chromosomes: *LcTPST1* is located on Chr3 and *LcTPST2* on Chr15; *LcPSK1* is tightly clustered with *LcCIF1-1* and *LcCIF1-2* on Chr13; and *LcPSK2* and *LcPSY1* are located on Chr6 and Chr18, respectively (Figure 5a).

Synteny analyses were performed using the genomes of *O. sativa*, *M. biondii*, *L. chinense*, *A. thaliana*, and *P. trichocarpa* to examine conserved genomic collinearity of *TPST*, *PSK*, *PSY*, and *CIF* loci across these species (Figure 5b). The results showed that *LcTPST1* is syntenic with the *TPST* members MBI26020 and MBI19822 in *M. biondii*, whereas no syntenic counterpart was detected in *A. thaliana*. In contrast, *LcTPST2* displayed synteny not only with *AtTPST* but also with *M. biondii TPST* members MBI26020 and MBI19822. *LcPSK1* showed syntenic relationships with MBI27034, *AtPSK2*, and the *P. trichocarpa* gene PNT02447. In addition to synteny with MBI27034 and *AtPSK2*, *LcPSK2* was also syntenic with *AtPSK3*, *AtPSK5*, PNT02447, and PNT26303. *LcCIF1-1* exhibited synteny only with MBI22811. No syntenic genes corresponding to *LcCIF1-2* or *LcPSY* were detected in *A. thaliana*, *M. biondii*, or *P. trichocarpa*. Moreover, no significant syntenic relationships were identified between any of the *L. chinense* genes examined and the corresponding *TPST*, *PSK*, *PSY*, or *CIF* family members in rice.

A collinearity comparison between *L. chinense* and the gymnosperm *Taxus chinensis* (Supplementary Figure S5) showed that only a limited number of syntenic blocks were detected between the two species, indicating an overall weak collinearity signal. In addition, no collinear gene pairs were identified for *TPST* or the sulfated peptide family. This pattern suggests that, following the long-term independent evolution of gymnosperms and angiosperms, microsyntenic conservation in the relevant genomic regions may have been substantially reduced, making it difficult to establish clear collinearity relationships for these two gene groups across distantly related lineages.

### 2.6. Molecular Docking

**Figure 6:**
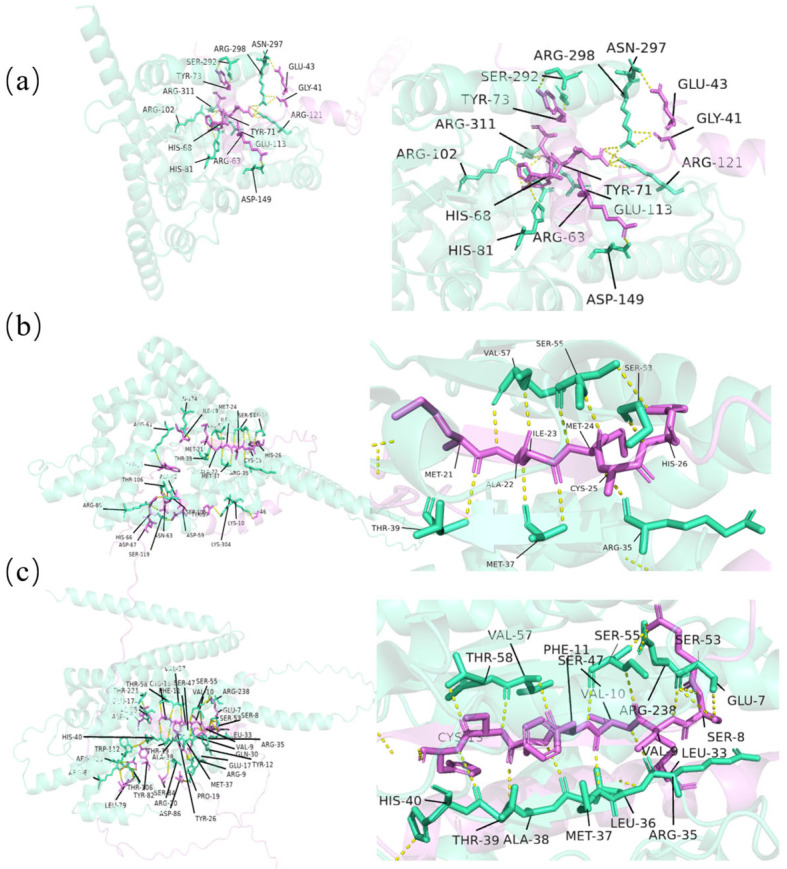
Protein–peptide docking models. (**a**) TPST–PSK. (**b**) TPST–PSY. (**c**) TPST–CIF. TPST is shown in green; PSK/PSY/CIF in purple; hydrogen bonds in yellow.

Three-dimensional structural analyses and molecular docking with interacting partners provided key mechanistic insights into the functions of LcTPST, LcPSK, LcPSY, and LcCIF (Figure 6). Docking of the LcTPST–LcPSK complex indicated that the major hydrogen-bond interactions are concentrated within the conserved sulfotransferase domain of LcTPST, involving HIS-81, ARG-102, GLU-113, ARG-121, and ASP-149, suggesting that these residues are critical for LcTPST-mediated sulfation of LcPSK. Notably, the docking models of LcTPST–LcPSY and LcTPST–LcCIF displayed a similar “sandwich-like” topology, i.e., an LcTPST–LcPSY/LcCIF–LcTPST conformation. The LcTPST interface residues were conserved between the two complexes and included ARG-35, MET-37, THR-39, SER-53, SER-55, and ALA-57, which are located near the N-terminus of the sulfotransferase domain. These observations collectively suggest that PSY and CIF may undergo comparable LcTPST-mediated sulfation modes.

### 2.7. Expression Patterns of Gene Family Members in L. chinense

**Figure 7:**
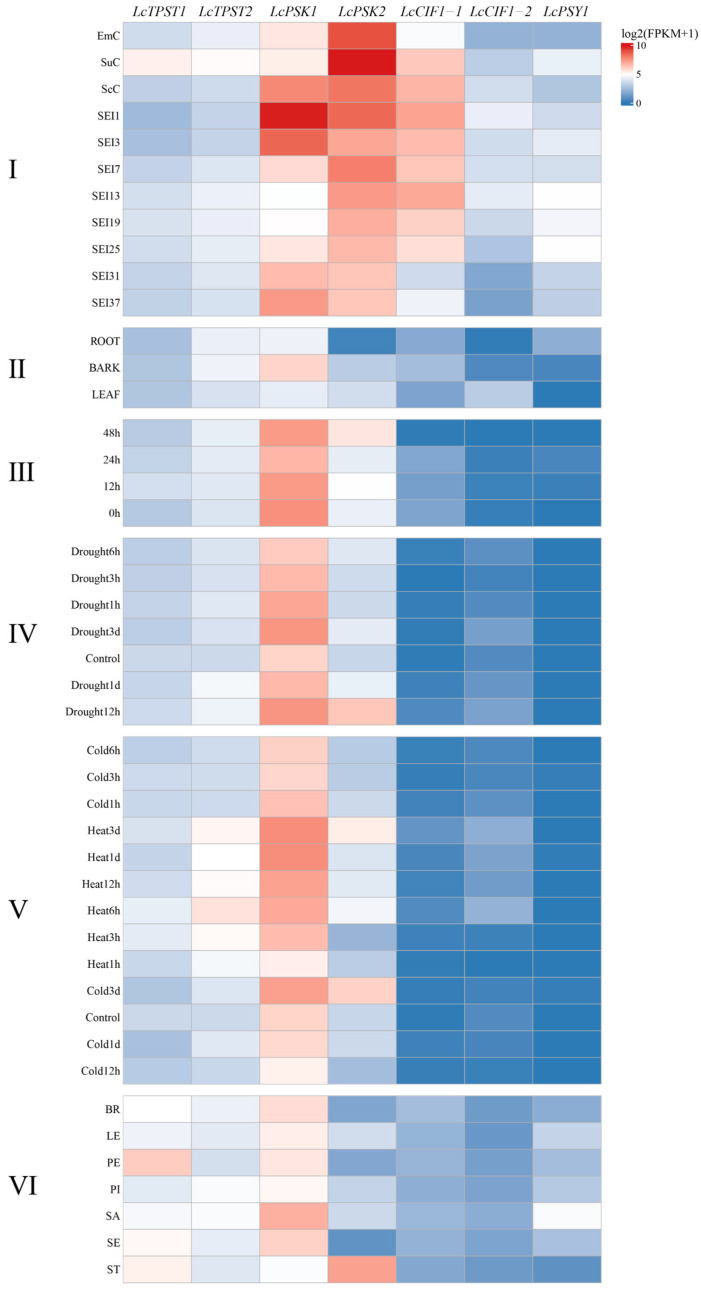
Expression profiles and stress responses of *Liriodendron TPST*, *PSK*, *PSY*, and *CIF* genes. (**I**) Somatic embryogenesis of *L. chinense*. EmC, embryogenic callus; SuC, suspension culture; ScC, single-cell culture; SEI1, 1 day after transfer to SEIM; SEI3, 3 days after transfer to SEIM; SEI7, globular embryo stage; SEI13, heart-shaped embryo stage; SEI19, torpedo embryo stage; SEI25, cotyledonary embryo stage; SEI31, mature cotyledonary embryo initiating germination; SEI37, plantlet stage. (**II**) Different organs of *L. chinense*. (**III**) Cold stress in *Liriodendron × sinoamericanum*. (**IV**) Drought stress in hybrid *Liriodendron*. (**V**) Temperature stress in *Liriodendron × sinoamericanum*. (**VI**) Different organs of *L. chinense*. BR, bract replicate; LE, leaf replicate; PE, petal replicate; PI, pistil replicate; SA, shoot apex replicate; SE, sepal replicate; ST, stamen.

In this study, publicly available RNA-seq datasets covering somatic embryogenesis, abiotic stress treatments, and organ development were integrated to systematically characterize the expression patterns of *LcTPST*, *LcPSK*, *LcPSY*, and *LcCIF* genes (Figure 7). Overall, *LcTPST* showed relatively low expression compared with sulfated peptide precursor genes. Among them, *LcTPST1* displayed relatively high expression in petal replicates (FPKM = 68.638), whereas *LcTPST2* exhibited higher mean expression under heat stress (FPKM = 23.985–46.988). *LcPSK1* and *LcPSK2* showed pronounced transcriptome-dependent differences: *LcPSK1* showed consistently high expression across six transcriptome datasets covering somatic embryogenesis, organ development, and abiotic stress responses, including cold, drought, and temperature stress treatments, whereas *LcPSK2* was highly expressed in stamen (FPKM = 141.958) and remained strongly expressed after the torpedo-embryo stage during somatic embryogenesis (FPKM > 150). *LcPSY* was expressed at extremely low levels under drought, cold, and heat treatments, but showed relatively higher expression at the cotyledonary embryo stage (FPKM = 29.304) and in shoot apex replicates (FPKM = 25.303). As a tandemly duplicated pair, *LcCIF1-1* and *LcCIF1-2* exhibited marked expression divergence: *LcCIF1-1* was relatively highly expressed mainly during somatic embryogenesis but remained extremely low under abiotic stress and in organ-development samples; *LcCIF1-2* also showed detectable expression primarily during somatic embryogenesis, yet its overall abundance was substantially lower than that of *LcCIF1-1*. Notably, at the single-cell culture stage, *LcCIF1-1* was expressed at a moderate level (FPKM = 25.392), whereas *LcCIF1-2* was barely detectable (FPKM = 3.353).

It should be noted that these integrated RNA-seq profiles represent bulk transcript abundance averaged across heterogeneous tissues and cell types. Therefore, the observed expression patterns may mask cell-type-specific or even opposite responses within the same organ under different developmental or stress conditions [27].

### 2.8. Co-Expression Networks and Transcription Factors

**Figure 8:**
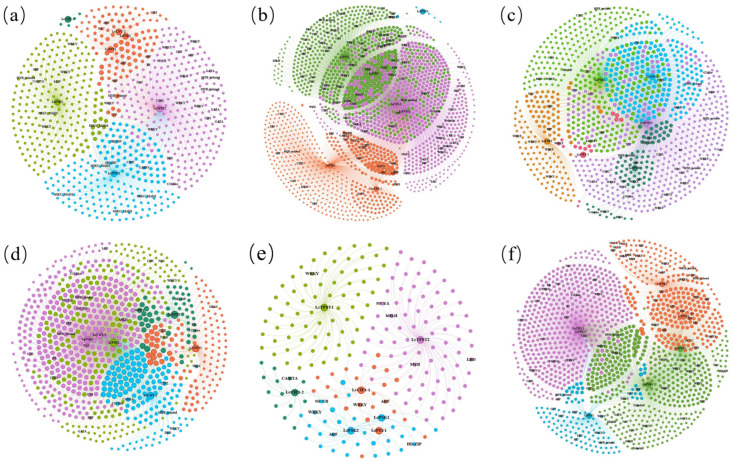
Co-expression network analysis of *Liriodendron TPST*, *PSK*, *PSY*, and *CIF* genes. (**a**) Different organs: bract replicate, leaf replicate, petal replicate, pistil replicate, shoot apex replicate, sepal replicate, and stamen. (**b**) Different organs: bark, leaf, and root. (**c**) Temperature stress: cold and heat stress. (**d**) Drought stress. (**e**) Cold stress. (**f**) Somatic embryogenesis. Each node represents a gene, and each edge indicates a co-expression relationship between two genes. Nodes shown in the same color within an individual panel represent the same co-expression module.

Focusing on *TPST* and its sulfated peptide substrates (*PSK*, *PSY*, and *CIF*) in *L*. *chinense*, we constructed gene co-expression networks across multiple transcriptome datasets spanning diverse organs, environmental stress treatments, and somatic embryogenesis. Overall, the networks comprised several highly connected hubs and dense submodules: *TPST* together with specific *PSK* members consistently occupied central positions across multiple contexts, suggesting coordinated transcription with a broad range of biological processes (Figure 8a–f).

Across the multi-organ expression dataset, node degrees of *TPST1*/*TPST2* and *PSK2* increased markedly, forming a dominant subnetwork. Transcription factors (TFs) showing strong co-expression were mainly enriched for MIKC_MADS (associated with floral organ differentiation), together with WRKY, bZIP, C2H2, and HD-ZIP families (Figure 8a). In the bark/leaf/root network, *PSK1* emerged as the largest hub, whereas *TPST1* was relatively peripheral; stress- and growth-related TFs, including WRKY, ERF, NAC, GATA, and LBD, were enriched around *PSK1* (Figure 8b). Under temperature stress, the interaction neighborhoods of *TPST2* and *PSK1* overlapped extensively and formed the network core, with multiple TF families involved, including WRKY, C2H2, bHLH, MYB-related, HD-ZIP, and ARF/TCP (Figure 8c). Drought treatment substantially increased overall network edge density and expanded the interwoven connectivity among *TPST* and *PSK/CIF* members (Figure 8d). Core TFs included WRKY, NAC, ERF, bHLH, C2H2, TALE, CO-like, GATA, and CAMTA. In the cold-only dataset, the network core further converged on the *PSK1*–*TPST2*–*CIF* module, accompanied by a marked reduction in the number of co-expressed genes; the enriched TFs were dominated by WRKY, bHLH, and ARF families (Figure 8e). During somatic embryogenesis, connectivity of *CIF* members with *TPST/PSK* was notably strengthened (Figure 8f), and development-associated TFs (WOX, LBD, YABBY, ARF, B3, and bHLH) were positioned close to the network center.

**Figure 9:**
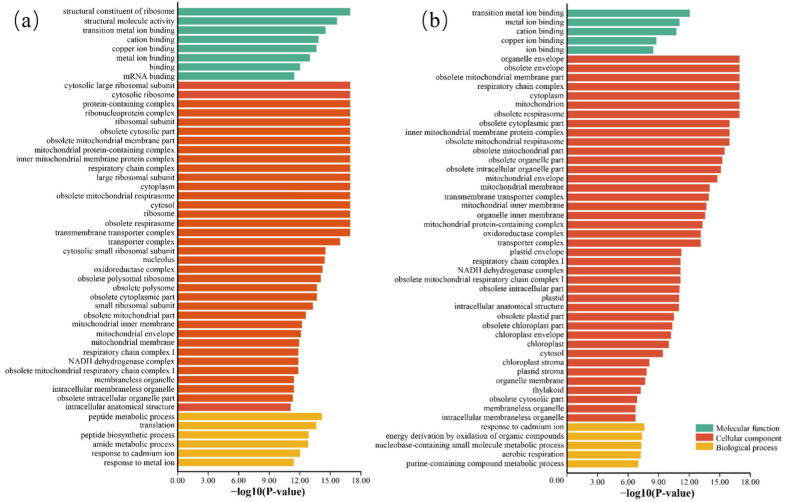
Gene Ontology enrichment analysis of genes co-expressed with TPST and sulfated peptide genes (PSK, PSY, and CIF) in *L. chinense*. (**a**) GO enrichment analysis of genes co-expressed with TPST and sulfated peptide genes during somatic embryogenesis. (**b**) GO enrichment analysis of genes co-expressed with TPST and sulfated peptide genes across different organs. BR, bract replicate; LE, leaf replicate; PE, petal replicate; PI, pistil replicate; SA, shoot apex replicate; SE, sepal replicate; ST, stamen.

To further characterize the functional features of these TF-associated co-expression modules, GO enrichment analysis was performed on representative co-expressed gene sets from the somatic embryogenesis and multi-organ transcriptomes (Figure 9). In the somatic embryogenesis dataset, the co-expressed genes were significantly enriched in translation, ribosome, peptide biosynthetic process, peptide metabolic process, and responses to metal ions (Figure 9a). In the multi-organ dataset, the co-expressed genes were mainly enriched in aerobic respiration, respiratory chain complex, mitochondrion, chloroplast, and other energy metabolism- or organelle-related categories (Figure 9b). These results indicate that the core TF-associated co-expression modules identified from representative developmental and organ-related datasets were functionally associated with protein synthesis, stress response, and energy metabolism.

## 3. Discussion

Since the first identification of *AtTPST* (At1g08030) in 2009, studies have demonstrated that this tyrosylprotein sulfotransferase can catalyze tyrosine sulfation of multiple peptide substrates, such as PSK and PSY1, in vitro [24]. Loss-of-function mutants exhibit prominent phenotypes, including dwarfism and impaired root growth, indicating that TPST is essential for the biological activity of sulfated peptide hormones [12,28]. Around the same period, multiple sulfated peptides were successfully identified, including PSK (1996) [29], PSY (2007) [19], RGF (2010) [5], and CIF (2017) [6], all of which play pivotal roles in regulating plant growth and development as well as stress responses [9]. However, the evolutionary history of the plant TPST-sulfated peptide system and its fine-tuned interaction mechanisms remain insufficiently and systematically elucidated.

Our analyses indicate that TPST is broadly maintained as a low-copy gene across diverse plant lineages and exhibits overall low transcript abundance across *L. chinense* transcriptomes. These observations suggest that, as an upstream enzyme responsible for activating multiple sulfated peptide hormones, TPST may act as a “shared rate-limiting factor” [30] whose function is highly conserved, thereby favoring retention at low copy number during evolution. By contrast, sulfated peptide precursor genes—more prone to local duplication or small-scale expansion-appear more likely to undergo copy-number increases, generating lineage-specific diversity in ligand composition and abundance.

Systematic mining of cross-lineage genomic datasets indicates that plant TPST is already present in early green plants such as chlorophytes, suggesting that the origin of this tyrosine-sulfation enzyme predates the emergence of land plants. In contrast, within the genomes surveyed here, the canonical TPST-dependent sulfated peptide families (PSK/PSY/CIF/RGF) were first detected only in the seed-plant lineage (beginning with gymnosperms), and no reliable homologous evidence was recovered from earlier-diverging lineages. This apparent temporal gap—“the enzyme preceding its substrates”—suggests that TPST may have processed other sulfated substrates before the emergence or substantial expansion of these classical peptide families (e.g., as-yet-unidentified peptide precursors or non-canonical secreted/membrane protein substrates) and that its functional repertoire may have been subsequently redirected as new peptide-signaling systems evolved. To further test this interpretation, we compared the conserved domains of TPST homologs across chlorophytes and higher plants. Only a few chlorophyte sequences retained a sulfotransferase-related domain (Sulfotransfer_2), whereas most TPST-like proteins in chlorophytes lacked canonical sulfotransferase domains and instead contained WW or PHD-related domains. In contrast, TPST proteins in higher plants generally possess the Sulfotransfer_1 domain associated with tyrosine sulfation activity. These results suggest that TPST-like proteins may have originated before the establishment of the canonical sulfated peptide system and subsequently underwent functional divergence and specialization during land plant evolution. It should be noted, however, that the failure to detect substrate peptides in early lineages may also reflect methodological and data-related limitations, including short precursor length, rapid sequence divergence, and incomplete genome annotation. Therefore, the specific functional roles of TPST in early lineages, as well as the extent to which these roles were retained, repurposed, or lost in later lineages, remain to be clarified through higher-quality genomic resources and direct experimental validation.

As an early diverging angiosperm, *L. chinense* provides an informative phylogenetic window for investigating the evolution of the sulfated peptide gene family (Supplementary Figure S1). Our comparative analysis showed that *L. chinense* contains an intermediate family size (seven members), exceeding the low-copy state observed in early land plants but remaining below the marked expansions found in several core eudicots (Supplementary Figure S2). In addition, orthogroup analysis indicated that *L. chinense* retained members in multiple conserved gene clusters, yet generally at low copy number and without obvious lineage-specific expansion (Supplementary Figure S3). These features suggest that *L. chinense* preserves a transitional evolutionary state in which the sulfated peptide system had already established its basic angiosperm framework but had not yet undergone the extensive expansion observed in later diverging lineages. Therefore, *L. chinense* represents a valuable model for tracing the early establishment and subsequent diversification of sulfated peptide signaling in angiosperms.

Among the sulfated peptide families identified in this study, PSY and RGF can be regarded as relatively “late-emerging” groups, with the earliest recognizable homologs recorded in basal angiosperms. Notably, although RGF homologs were detected in two Lauraceae species, no RGF family members were identified in monocots, a lineage often considered more evolutionarily derived. To account for this unusual distribution pattern, we propose two non-mutually exclusive explanations. First, RGF may have originated early in angiosperm evolution and subsequently expanded in eudicots but undergone widespread loss in the monocot lineage, potentially reflecting shared physiological or developmental requirements between Lauraceae and eudicots. Second, because RGF precursor peptides are short and the mature peptide region may evolve rapidly, homolog detection across distantly related lineages may be challenging, leading to missed annotations or biases in currently available genome resources.

The biological outputs of sulfated peptide signaling are shaped not only by the emergence and expansion of peptide families but also by receptor recognition specificity, assembly of receptor/co-receptor complexes, and the efficiency of downstream signal transduction. Current evidence indicates that sulfated peptides such as PSK, PSY, CIF, and RGF are generally perceived by plasma membrane–localized leucine-rich repeat receptor kinases (LRR-RKs) [11,31,32], and each pathway exhibits relatively well-defined ligand–receptor pairings (e.g., PSK–PSKR [11], CIF–SGN3/GSO1 and GSO2 [33], and RGF–RGFR/RGI [34]), often with members of the SERK family acting as co-receptors to promote receptor activation and signal amplification [33,34,35]. Structural and quantitative binding studies further demonstrate that the sulfotyrosine (sTyr) generated by TPST frequently contributes directly to the ligand–receptor ectodomain interface, thereby markedly enhancing binding affinity between sulfated peptides and their cognate LRR-RK receptors. For instance, in the CIF–GSO1/SGN3 system, desulfation reduces affinity by~100–1000-fold [33], and comparable decreases of~200-fold [36] and~25-fold have been reported for the RGF and PSK systems, respectively.

During PSK recognition, the crystal structure of the AtPSKR1-PSK complex (PDB: 4Z63) shows that the two sulfotyrosines in PSK (chain P; TYS28-ILE29-TYS30-THR31-GLN32)-sTyr1 (TYS28) and sTyr3 (TYS30)-serve as the principal recognition anchors [37]. Their sulfate groups form a critical hydrogen-bond/salt-bridge network primarily with Lys508 at the receptor binding interface (chain A), assisted by other polar or charged residues such as Asn424, Glu511, and Arg349. Meanwhile, the ligand backbone packs against the island-domain β-strand composed of Phe503-Pro504-Phe505-Phe506-Met507-Lys508-Arg509-Asn510–Glu511, forming a stable antiparallel β-sheet [37]. This architecture enables high-affinity binding and induces allosteric rearrangements of the receptor ectodomain, thereby promoting receptor activation and recruitment of SERK co-receptors [38]. In *L. chinense*, we likewise observed a recognition geometry consistent with that of the AtPSK–AtPSKR1 complex: the sulfate groups of the two sulfotyrosines in PSK (sTyr; TYS72/TYS74) act as key electrostatic anchors that govern ligand positioning and stabilization within the receptor-binding pocket, suggesting that this ligand–receptor recognition framework is highly conserved evolutionarily. Nevertheless, whether *L. chinense* PSKR further recruits SERK co-receptors upon ligand binding to assemble an activated receptor complex remains to be validated through co-immunoprecipitation and in vivo interaction assays, structural determination, and/or genetic evidence (Supplementary Figure S4).

In *L. chinense*, we identified two *TPST* copies, two *PSK* copies, one *PSY*, and two tandemly duplicated *CIF* genes. The promoter regions of all seven family members were generally enriched for anaerobic induction- and abscisic acid (ABA)-responsive *cis*-elements, suggesting that the TPST-sulfated peptide system may broadly participate in hypoxia-related stress responses and ABA-mediated signaling pathways. With the exception of *LcPSK2* and *LcPSY*, the promoters of the remaining five genes contained methyl jasmonate (MeJA)-responsive elements, implying potential roles in defense- or stress-related pathways. Notably, only *LcPSK2* showed specific enrichment of *cis*-elements associated with seed-specific regulation, palisade mesophyll cell differentiation, and endosperm expression, indicating that it may play a more specialized regulatory role in seed development and leaf tissue differentiation. The two tandemly duplicated *CIF* genes exhibited overall similar *cis*-element types and copy numbers; however, *LcCIF1-1* additionally harbored nine circadian control–related elements. This divergence likely reflects de novo insertional changes in the promoter after gene duplication and/or differences in local chromatin context, thereby conferring potential regulatory differentiation in circadian-dependent expression.

TPST, PSK, PSY, and CIF family members in *L. chinense* generally contain hormone and stress-responsive *cis*-elements, suggesting that they may collectively participate in the regulation of growth, development, and environmental adaptation. It should be noted, however, that *cis*-elements indicate only potential regulatory capacity, and their actual functions are strongly dependent on cell type and developmental context; even within the same organ, different cell populations may respond differently to the same signal. Therefore, these elements cannot be simply interpreted as evidence of uniform responses at the whole organ level. Differences in *cis*-element composition among family members further suggest regulatory partitioning and functional diversification.

Based on AlphaFold-based structural predictions and PyMOL-assisted docking analyses, the binding mode of LcTPST with LcPSK differed markedly from its docking patterns with LcPSY and LcCIF. In particular, interactions of LcTPST with LcPSY or LcCIF formed a distinctive yet highly consistent “sandwich-like” interface topology, a feature that may be associated with the physicochemical properties of PSY and CIF mature peptides, which are enriched in basic residues. Accordingly, we propose that LcPSY and LcCIF may follow similar recognition and catalytic modes during LcTPST-mediated sulfation. Notably, no RGF homologs were detected in the current *L. chinense* genome or transcriptome datasets; therefore, the molecular details of the TPST–RGF interaction interface remain to be resolved in other plant systems.

Previous studies have shown that TPST substrate recognition is closely associated with the local sequence environment surrounding the target tyrosine residue, with a preference for acidic residue-enriched regions. In particular, the N-terminal Asp–Tyr (DY) motif is regarded as an important feature for tyrosine sulfation in plant peptide precursors, and the presence of additional Asp/Glu residues around the target Tyr can further enhance TPST recognition and sulfation efficiency [24]. This feature is conserved in multiple classes of plant sulfated peptide precursors: PSY family precursors generally retain motifs related to tyrosine sulfation, members of the RGF/GLV/CLEL family share the characteristic Asp–Tyr signature also found in PSK and PSY, and CIF peptides contain an N-terminal Asp–Tyr motif required for sulfation [2,23,33,39]. Taken together, these observations suggest that TPST recognition of different sulfated peptide precursors may rely more on shared local sequence determinants than on the overall architecture of the peptide substrate.

Against the background of consistently low *TPST* expression across transcriptome datasets, *LcPSK1* displayed broad and stable high expression during somatic embryogenesis, under cold/heat and drought stresses, and across multiple vegetative and reproductive organs, suggesting a “generalist” role as a core regulator of growth, development, and environmental adaptation in *L. chinense*. In contrast, *LcPSK2* exhibited pronounced upregulation primarily in somatic embryogenesis samples, indicating functional specialization and division of labor among members of the same gene family across developmental processes. The two tandemly duplicated *CIF* genes showed strikingly divergent expression during somatic embryogenesis; this divergence is difficult to explain solely by differences in promoter *cis*-element composition and is more likely attributable to epigenetic variation, such as differences in DNA methylation, histone modifications, or chromatin accessibility. These observations provide a rationale for future validation of their regulatory divergence at the epigenomic level.

## 4. Materials and Methods

### 4.1. Identification and Physicochemical Characterization of Five Gene Families

A total of 126 plant species were included in this study, comprising 60 chlorophytes, 3 bryophytes, 4 ferns, 9 gymnosperms, 8 basal angiosperms, 9 monocots, and 33 eudicots. Genome resources were retrieved from the NCBI database [40], Phytozome [41], and the Genome Warehouse of the National Genomics Data Center, China National Center for Bioinformation [42]. Full-length AtTPST, AtPSK, AtPSY, AtCIF, and AtRGF sequences were used as queries. Candidate homologs were identified by BLASTP (v2.5.0+)searches (*E*-value < 1 × 10^−5^) [43] and hidden Markov model (HMMER 3.3)-based searches against the predicted proteomes of each species to obtain best hits while minimizing false positives [44]. Domain prediction was performed using the NCBI Conserved Domain Database to confirm assignment to the corresponding protein families [45,46]. For PSK, PSY, CIF, and RGF precursor proteins, sequences longer than 200 amino acids were excluded, and the remaining sequences were retained as candidate PSK, PSY, CIF, and RGF members.

The physicochemical properties of the identified proteins—including amino acid length, molecular weight (MW), theoretical isoelectric point (pI), instability index, aliphatic index, and grand average of hydropathicity (GRAVY)—were calculated using Tbtools-II (v2.323) [47].

### 4.2. Phylogenetic Analysis

A species phylogeny of the 66 non-algal taxa was generated using the online TimeTree resource to depict the evolutionary relationships among the selected species [48]. For phylogenetic analyses of TPST, PSK, PSY, CIF, and RGF proteins, sequences from the 126 species were aligned by multiple sequence alignment using DNAMAN (v6.0.3.99). Neighbor-joining (NJ) trees were then constructed in MEGA11 [49]. To ensure phylogenetic robustness, maximum-likelihood trees were inferred with IQ-TREE (v2.0.3) using the best-fitting substitution model selected by ModelFinder [50]. All trees were visualized and esthetically refined using ChiPlot tvBOT (version 2.6.1).

### 4.3. Cis-Element Analysis, Chromosomal Mapping, and Synteny Analysis

Using TBtools-II, 2000 bp upstream sequences from the ATG start codon of *LcTPST*, *LcPSK*, *LcPSY*, and *LcCIF* were extracted. *Cis*-regulatory elements (CREs) were predicted using the PlantCARE database [51], and the results were visualized and esthetically refined in R (v4.4.3). Chromosomal locations of *LcTPST*, *LcPSK*, *LcPSY*, and *LcCIF* genes were plotted in TBtools-II. Synteny analyses and visualization were performed using MCScanX and the Multiple Synteny Plot functions implemented in TBtools-II [52].

### 4.4. Molecular Docking of Proteins

The three-dimensional structures of LcTPST in complex with LcPSK/LcPSY/LcCIF were predicted using the online AlphaFold server [53], with the automatic modeling mode selected in the AlphaFold analysis interface. The predicted 3D structures were visualized using PyMOL (v3.10).

### 4.5. Transcriptome Analysis

RNA-seq datasets for *Liriodendron* were retrieved from the NCBI Sequence Read Archive (SRA), including transcriptomes from different organs of *L. chinense* (PRJNA559687 and PRJNA780974) [54,55], as well as datasets from the *Liriodendron.chinense* × *Liriodendron.tulipifera* covering somatic embryogenesis, cold/heat stress (PRJNA1177383 and PRJNA679089) [56], drought stress (PRJNA679101) [56], and cold stress (PRJNA761222). Expression profiles of target genes were extracted and visualized in R.

### 4.6. Gene Co-Expression Network Analysis

The co-expression correlation analysis tool on the OEBiotech platform (Shanghai, China) was used to calculate Pearson’s correlation coefficients (PCCs) between *LcTPST*, *LcPSK*, *LcPSY*, *LcCIF*, and other genes. Genes meeting the thresholds of |PCC| ≥ 0.60 and adjusted *p* values (Adj. *p*) < 0.05 were retained as significantly co-expressed partners and considered potential regulatory candidates. The resulting network was visualized using Gephi (v0.10.1) [57].

### 4.7. Gene Ontology Enrichment Analysis

The gene sets significantly co-expressed with the target genes were used as the query lists, and the species-specific GO annotation file was used as the background annotation set for Gene Ontology (GO) enrichment analysis. GO enrichment analysis was performed in R using the clusterProfiler package, and enrichment was evaluated separately for the Biological Process (BP), Cellular Component (CC), and Molecular Function (MF) categories. Statistical significance was assessed using the hypergeometric test, and *p* values were adjusted for multiple testing using the Benjamini–Hochberg method to control the false discovery rate (FDR). GO terms with an adjusted *p* value (FDR) < 0.05 were considered significantly enriched. Enrichment results were visualized using barplot, dotplot, and customized plotting functions.

## Data Availability

The transcriptomic data used in this study are available from the NCBI Sequence Read Archive (SRA) under accession numbers PRJNA559687, PRJNA780974, PRJNA1177383, PRJNA679089, PRJNA679101 and PRJNA761222. The data were previously published and can be accessed via the repository.

## Supplementary Materials

The following supporting information can be downloaded at: https://www.mdpi.com/article/10.3390/plants15071115/s1, Figure S1: Phylogenetic framework and divergence timeline of the sulfation gene family across representative plant species; Figure S2: Gene family size distribution and evolutionary trend of sulfation-related genes across plant species; Figure S3: Orthogroup distribution of sulfation gene family members across representative plant species; Figure S4: Protein–peptide docking models of LcPSK-LcPSKR; Figure S5: Collinearity analysis between *Liriodendron chinense* and *Taxus chinensis*; Table S1: TPST protein quantity identified in Chlorophyta species.
