# Supplementary material for: Evolutionary and Transcriptomic Analyses of the Plant TPST-Sulfated Peptides System, with Insights from Woody Liriodendron chinense
Source: Plants (Basel). 2026 Apr 4;15(7):1115. doi: 10.3390/plants15071115 (PMC13074997; doi:10.3390/plants15071115)
Supplement: Supplementary file 1 [file plants-15-01115-s001.zip › Supplementary Files/Supplementary Figure .docx]

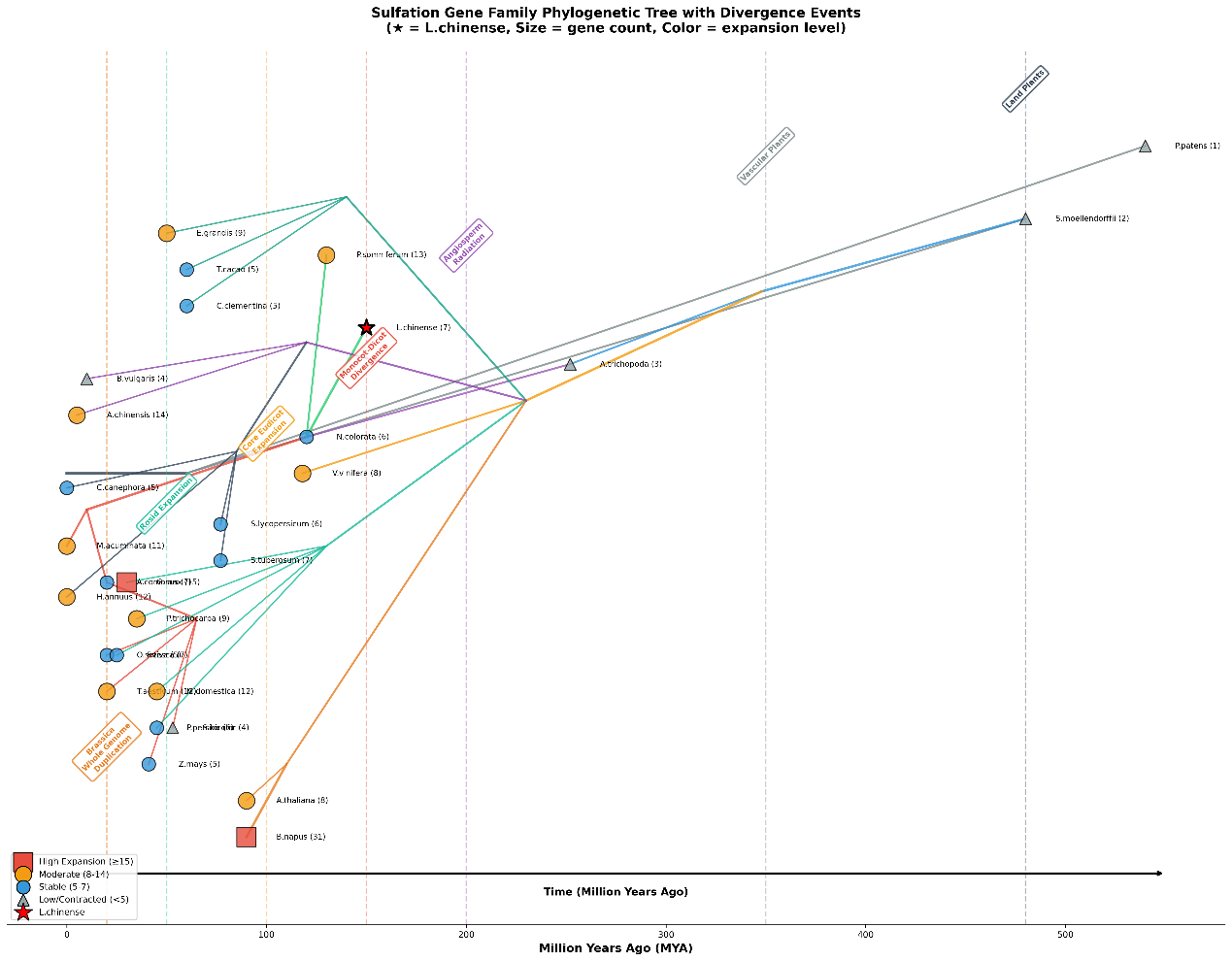


**Supplementary Fig. 1** Phylogenetic framework and divergence timeline of the sulfation gene family across representative plant species.


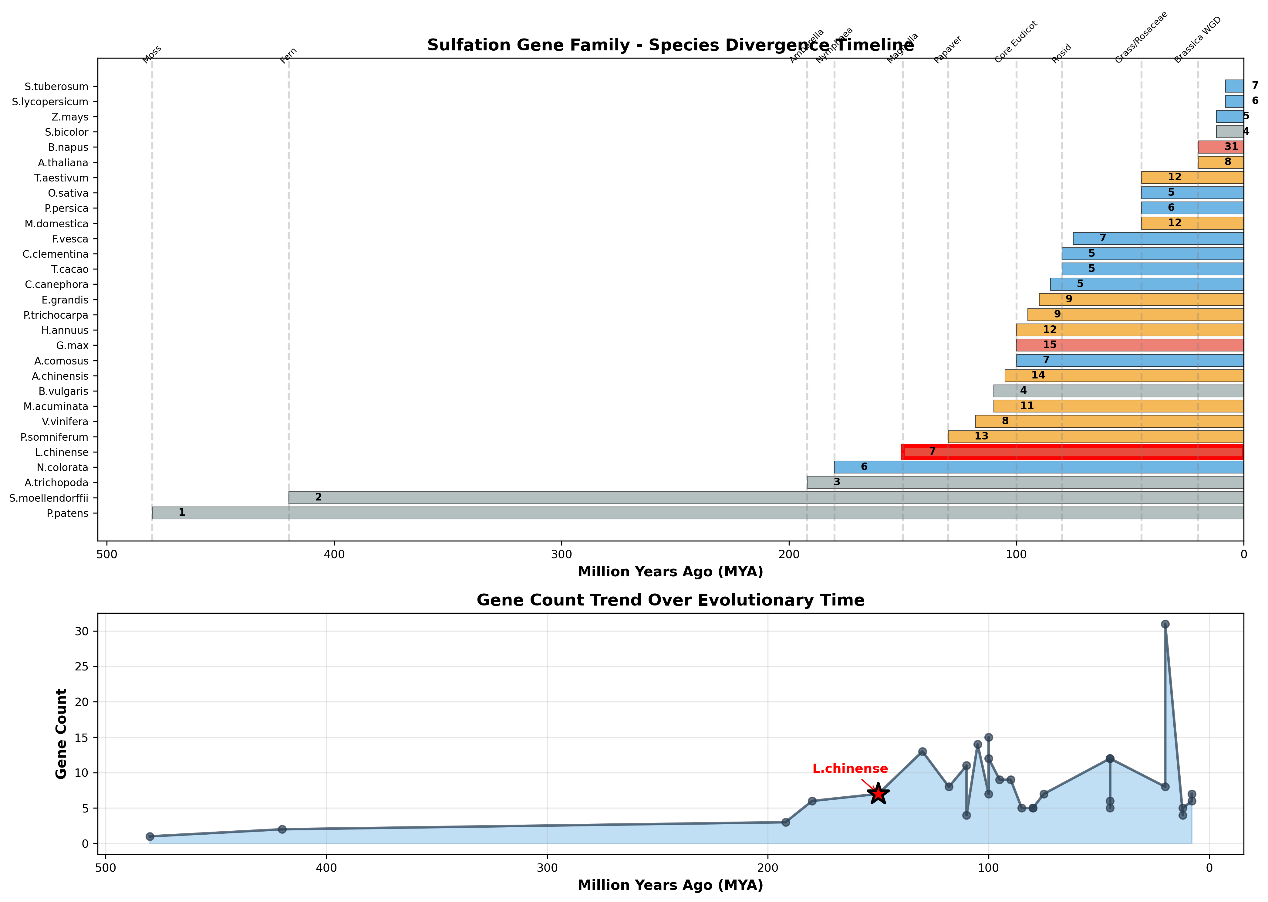


**Supplementary Fig. 2** Gene family size distribution and evolutionary trend of sulfation-related genes across plant species.
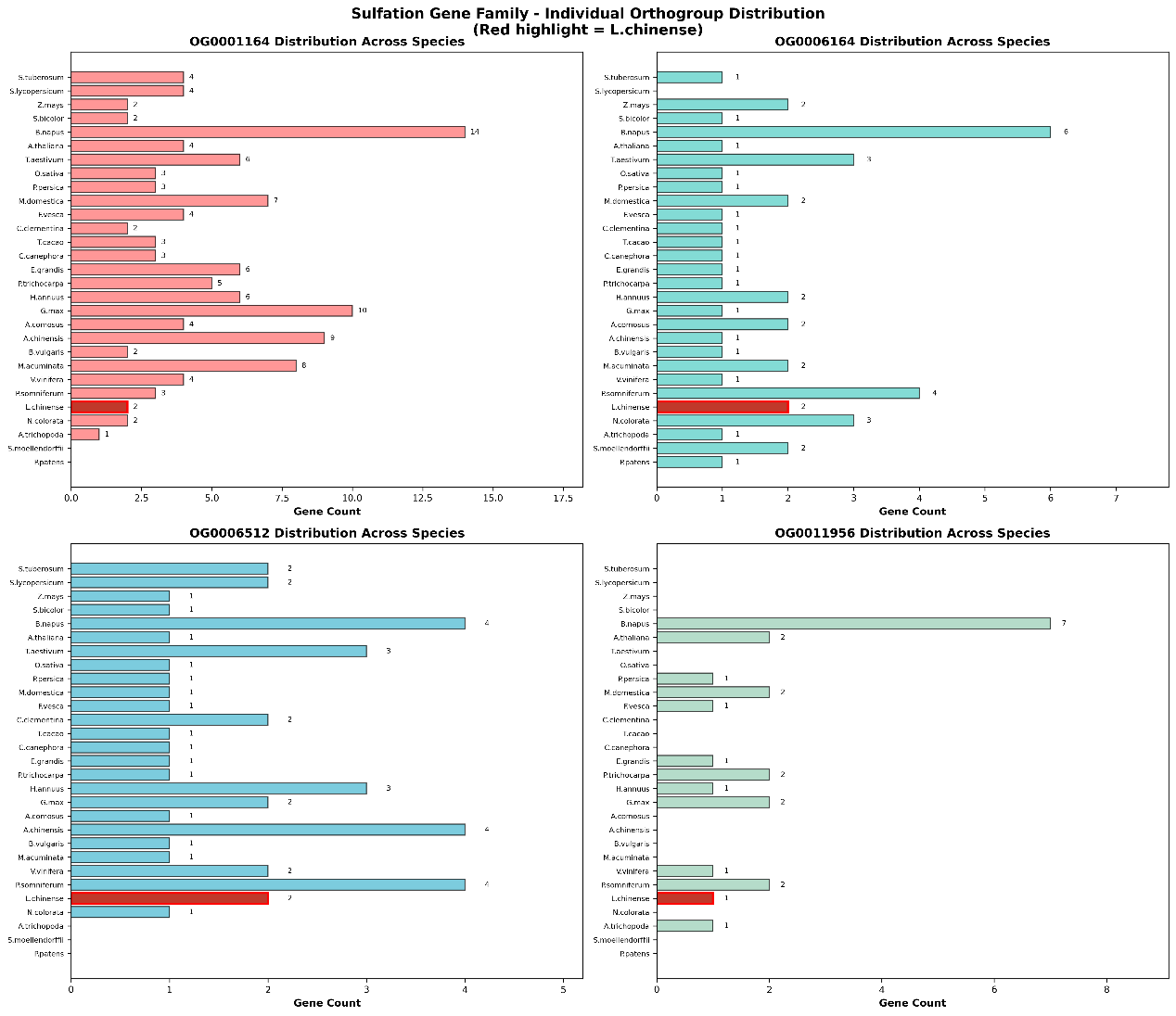


**Supplementary Fig. 3** Orthogroup distribution of sulfation gene family members across representative plant species.


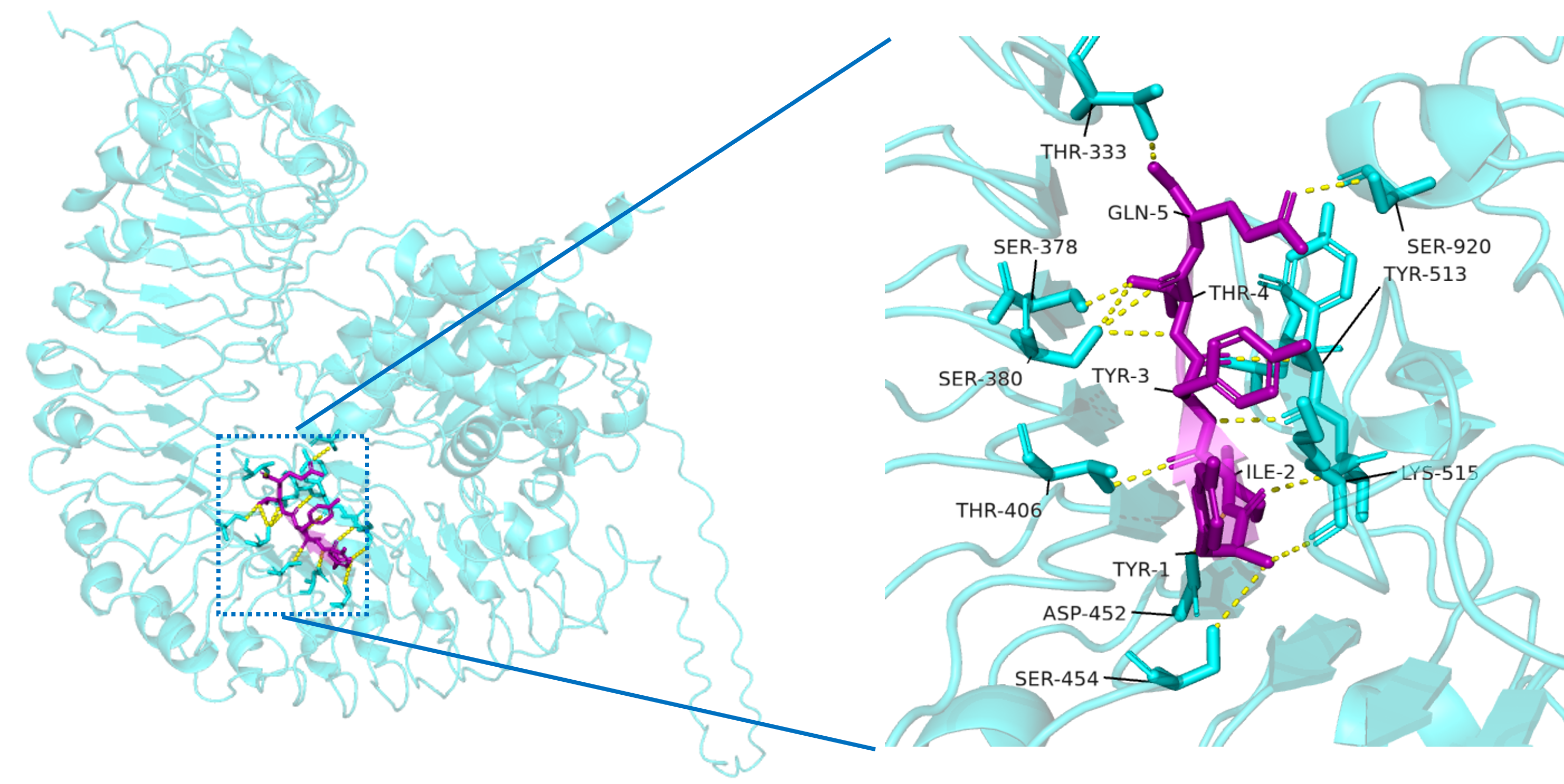


**Supplementary Fig. 4** Protein–peptide docking models of LcPSK-LcPSKR. PSKR is shown in blue; PSK in purple; hydrogen bonds in yellow.


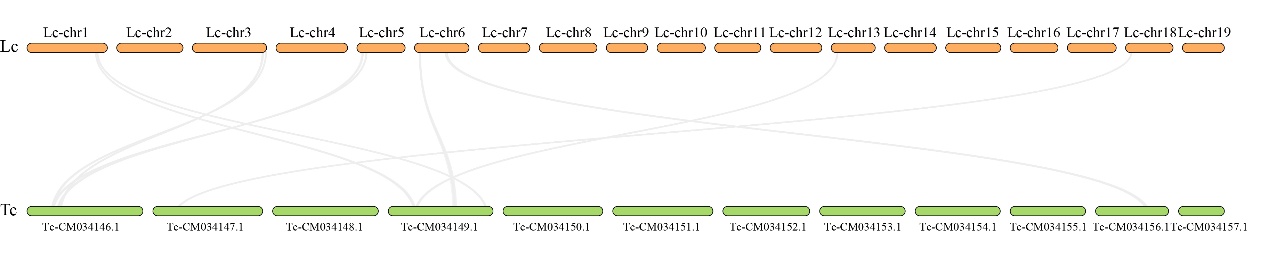


**Supplementary Fig. 5** Collinearity analysis between *Liriodendron chinense* and *Taxus chinensis*.
